# Serum VEGF levels as predictive marker of bisphosphonate-related osteonecrosis of the jaw

**DOI:** 10.1186/1756-8722-5-56

**Published:** 2012-09-17

**Authors:** Bruno Vincenzi, Andrea Napolitano, Alice Zoccoli, Michele Iuliani, Francesco Pantano, Nicola Papapietro, Vincenzo Denaro, Daniele Santini, Giuseppe Tonini

**Affiliations:** 1 Department of Medical Oncology, Campus Bio-Medico University of Rome, Rome, Italy; 2 Department of Orthopaedic and Trauma Surgery, Campus Bio-Medico University of Rome, Rome, Italy

**Keywords:** Osteonecrosis of the jaw, Bisphosphonates, Vascular endothelial growth factor (VEGF)

## Abstract

Recent studies have been reported that angiogenesis suppression may play a role in developing bisphosphonate-related osteonecrosis of the jaw (B-ONJ). According to these evidence we evaluated the role of VEGF as predictive marker of B-ONJ onset. Of the 81 patients, 6 developed B-ONJ following bisphosphonate treatment. These patients showed a strongest decrease in VEGF circulating levels at day 7 and at day 21 after the first administration. These data demonstrated for the first time that the anti-angiogenic properties of bisphosphonates are directly linked to B-ONJ pathogenesis and serum VEGF levels could represent an effective early predictive marker.

## Letter to the editor

Bisphosphonate-related osteonecrosis of the jaw (B-ONJ) is defined as non-healing exposed bone in the mandible or maxilla persisting for more than 8 weeks in a patient with no history of radiation therapy on the jaws who has taken or is currently taking a bisphosphonate. B-ONJ is a well-known adverse effect reported in about 5% of patients with cancer receiving high-dose intravenous bisphosphonates [1].

B-ONJ pathogenesis is still uncertain and under debate; bisphosphonates indeed act on a wide variety of cells, possibly increasing susceptibility to infections in the oral cavity and impairing mucosal healing acting on monocyte differentiation, angiogenesis or both [1].

Recently, several cases of exposed bone in the mandible, with a very close clinical presentation to B-ONJ, have been reported in cancer patients treated with bevacizumab, a recombinant human monoclonal antibody that binds to vascular endothelial growth factor (VEGF) and inhibits angiogenesis [2,3]. According to popular hypothesis, angiogenesis suppression may indeed play a role in B-ONJ [4], even though evidences are currently lacking. Notably, vascular impairment is considered to be crucial in the pathogenesis of other bisphosphonate-unrelated osteonecrosis (e.g. osteoradionecrosis, avascular necrosis of the hip and corticosteroid-induced osteonecrosis).

Notably, beside known antiresorptive, immunomodulating and direct antitumor actions, bisphosphonates are now considered to be also anti-angiogenic drugs: in our three previous works we showed the reduction of circulating VEGF in cancer patients suffering from bone metastases after treatment with either zoledronic acid (standard schedule or metronomic administration) or pamidronate [5-7].

## Findings

In order to evaluate the role of VEGF reduction as predictive marker of B-ONJ onset, we collected data from our three series involving a total of 81 patients. Of 81 patients, 6 (7.4%) developed B-ONJ following intravenous bisphosphonate administration (1 under treatment with pamidronate, 3 under treatment with zoledronic acid on standard schedule and 2 under treatment with zoledronic acid on metronomic administration). These 6 patients were affected by breast cancer (n = 3), prostate cancer (n = 2) and renal cancer (n = 1). The median number of bisphosphonates administrations was 9 in B-ONJ group vs. 11 in non-B-ONJ group (p = 0.196) and the median time of B-ONJ development was 9 months (range 6–22).

Compared to the patients who didn’t develop B-ONJ, these 6 patients showed the strongest decrease in VEGF circulating levels at day 7 (median = 540.81, CI95% 129.35-634.64 vs. median = 788.55, CI95% 728.96-870.44, P < 0.0001) and at day 21 (median = 458.00, CI95% 369.05-601.29 vs. median = 710.81, CI95% 638.66-955.53, P < 0.0001) after the first administration of either zoledronic acid or pamidronate. VEGF levels of the 6 patients are summarized in Table 1. VEGF was assayed using the R&D quantitative kit according to the manufacturer's instructions (R&D Systems, Minneapolis, MN). The detection limit of the VEGF was 62.5 pg/ml.

**Table 1:** VEGF levels (pg/ml) at baseline, 7 and 21 days in patients who developed B-ONJ

	**VEGF levels (pg/ml)**		
**Patients**	***baseline***	***7 days***	***21 days***
#1	804,66	407,82	350,00
#2	995,36	456,97	433,45
#3	970,44	510,11	473,00
#4	847,00	705,09	799,03
#5	932,00	564,23	468,00
#6	883,60	590,70	603,00

To the best of our knowledge, this is the first evidence that the anti-angiogenic properties of bisphosphonates are directly linked to B-ONJ pathogenesis. If confirmed, serum VEGF levels at day 7 and 21 after the first administration of intravenous bisphosphonate could represent an effective early predictive marker of B-ONJ.Finally, we are aware that these data come from a very small retrospective analysis, but their potential impact in clinical practice warrants prospective studies and extensive validation by the entire medical community.

## Abbreviations

B-ONJ: Bisphosphonate-related osteonecrosis of the jaw; VEGF: Vascular endothelial growth factor.

## Competing interests

The authors declare that they have no competing interests.

## Authors' contributions

**BV** conceived the study and gave substantial contribution to data analysis and interpretation; **AN** have been involved in drafting and revising the manuscript; **AZ**, **MI** and **FP** have been involved in the acquisition, analysis and interpretation of data; **DS**, **NP**, **VD** and **GT** have been involved in revising critically the manuscript. All authors read and approved the final manuscript.
